# MiR&moRe2: A Bioinformatics Tool to Characterize microRNAs and microRNA-Offset RNAs from Small RNA-Seq Data

**DOI:** 10.3390/ijms21051754

**Published:** 2020-03-04

**Authors:** Enrico Gaffo, Michele Bortolomeazzi, Andrea Bisognin, Piero Di Battista, Federica Lovisa, Lara Mussolin, Stefania Bortoluzzi

**Affiliations:** 1 Department of Molecular Medicine, University of Padova, 35121 Padova, Italy; michele.bortolomeazzi@gmail.com (M.B.); andrea.bisognin@gmail.com (A.B.); 2 Division of Pediatric Hematology, Department of Women’s and Children’s Health, University of Padova, 35128 Padova, Italy; piero.dibattista@phd.unipd.it (P.D.B.); federica.lovisa@unipd.it (F.L.); lara.mussolin@unipd.it (L.M.); 3 Istituto di Ricerca Pediatrica Città della Speranza, 35127 Padova, Italy; 4 Interdepartmental Research Center for Innovative Biotechnologies (CRIBI), University of Padova, 35131 Padova, Italy

**Keywords:** miRNAs, moRNAs, isomiRs, isomoRs, small RNA prediction, bioinformatics, non-coding RNAs

## Abstract

MicroRNA-offset RNAs (moRNAs) are microRNA-like small RNAs generated by microRNA precursors. To date, little is known about moRNAs and bioinformatics tools to inspect their expression are still missing. We developed miR&moRe2, the first bioinformatics method to consistently characterize microRNAs, moRNAs, and their isoforms from small RNA sequencing data. To illustrate miR&moRe2 discovery power, we applied it to several published datasets. MoRNAs identified by miR&moRe2 were in agreement with previous research findings. Moreover, we observed that moRNAs and new microRNAs predicted by miR&moRe2 were downregulated upon the silencing of the microRNA-biogenesis pathway. Further, in a sizeable dataset of human blood cell populations, tens of novel miRNAs and moRNAs were discovered, some of them with significantly varied expression levels among the cell types. Results demonstrate that miR&moRe2 is a valid tool for a comprehensive study of small RNAs generated from microRNA precursors and could help to investigate their biogenesis and function.

## 1. Introduction

MicroRNAs (miRNAs) are ~22 nt non-coding transcripts playing a significant role in gene expression regulation [1]. In the last few years, thousands of novel miRNAs have been discovered by bioinformatics analysis of small RNA (sRNA) deep sequencing (sRNA-seq) data [2]. Moreover, analysis of sRNA-seq data brought to the discovery of microRNA-offset RNAs (moRNAs), a class of miRNA-sized RNAs that arise from pre-miRNA proximal regions [3]. Furthermore, high-throughput RNA sequencing technology (RNA-seq), by the possibility of inspecting sRNA sequences with nucleotide resolution, enabled the identification of sRNA variants (isomiRs), which can diversify miRNA function through modulation of miRNA-target recognition [4].

Since their discovery, moRNAs have been detected in different organisms [3,5,6,7,8,9,10], including humans [11,12,13,14,15,16,17]. MoRNAs are characterized by high sequence conservation [18] and have been demonstrated to be functional RNAs [14], potentially having an miRNA-like regulative effect [5]. Moreover, it has been shown that moRNA expression can vary between different phenotypes in mammals [19] and is deregulated in human disease [15,17]. Although moRNA biogenesis has not yet been fully elucidated, it has been hypothesized that moRNAs may originate by alternative Drosha/DGCR8-mediated processing of the pre-miRNA hairpins [15,20]. Besides, moRNA expression can be independent from, or compete with, the adjacent miRNA [14,21].

To detect moRNAs, most authors had to devise custom bioinformatics procedures that analyzed the sRNA-seq data left uncharacterized by the existing software tools. To date, no tool that explicitly characterizes moRNAs, miRNAs, and isomiRs from sRNA-seq data is publicly and freely available to the scientific community [2]. To fill this gap, we improved our method, which was successfully used to disclose and study miRNA-like RNAs [8,13], to develop and publicly release miR&moRe2. 

This manuscript describes the miR&moRe2 pipeline with a series of tests based on six datasets. First, miR&moRe2 results are compared with findings from three previous moRNA studies, to show that miR&moRe2 can detect previously described moRNAs. Then, miR&moRe2 predictions were validated considering data of small RNA expression upon miRNA biogenesis pathway knockdown. Finally, to illustrate miR&moRe2 usefulness and discovery power, miR&moRe2 was applied to a large sRNA-seq dataset in which moRNAs were previously not investigated.

## 2. Results

### 2.1. The MiR&moRe2 Software Pipeline

MiR&moRe2 is a bioinformatics software tool that implements different steps of sRNA-seq data processing by linking several custom scripts written mainly in Python, R and Bash, as well as available bioinformatics tools (see Methods).

In the current implementation, miR&moRe2 requires as minimal input a dataset of reads in FASTQ format from Illumina sRNA-seq, the reference genome of the organism under study in FASTA format, and possibly, gene annotations of the known mature miRNAs and miRNA precursors, in GFF3 format. MiRNA annotation can be retrieved from miRBase database [22] and reference genomes are available from UCSC [23] (https://genome.ucsc.edu/) or Ensembl [24] (https://www.ensembl.org). 

**Figure 1:**
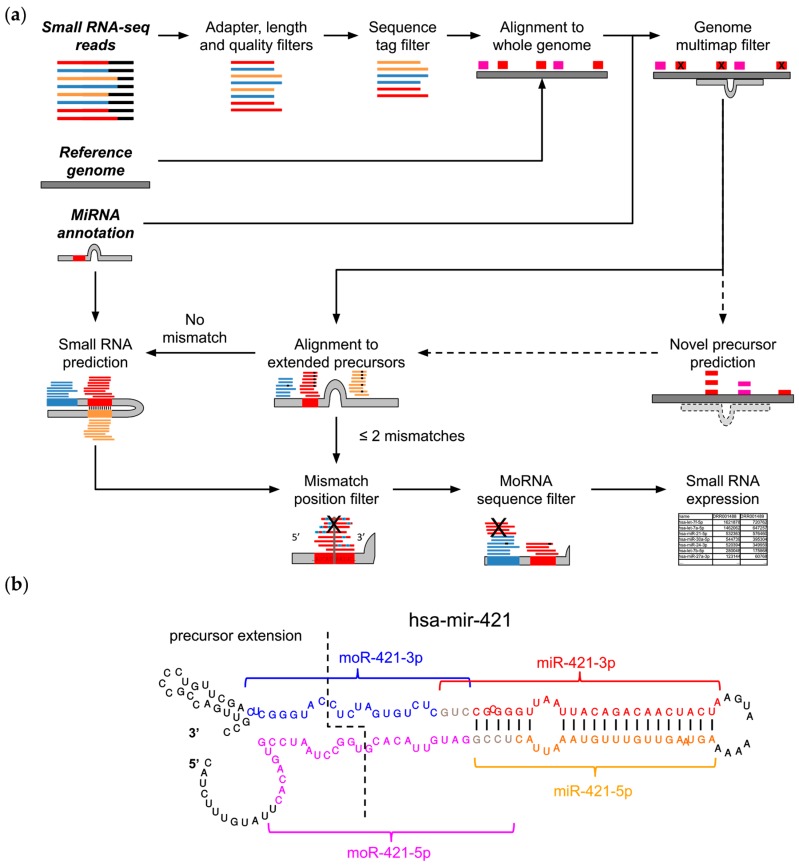
MiR&moRe2 features. (**a**) The miR&moRe2 pipeline workflow for sRNA data processing. Input data on the top left is highlighted by bold italic text. Dashed arrows indicate optional processing steps, bars represent adapter (black) and sRNA sequences: known miRNAs (red), unknown complementary miRNAs (orange) and moRNAs (blue). The extended miRNA precursor and its folding is represented in grey, along with vertical black bars indicating the complementary bases for novel miRNA prediction; (**b**) Secondary structure of the hsa-mir-421 miRNA precursor hairpin, with extended nucleotides at both ends (left side of the dashed line). Colored fonts and curly brackets indicate the positions of the annotated miR-421-3p (red font), and the three sRNAs predicted by miR&moRe2: the complementary miRNA miR-421-5p (orange font), moR-421-3p (blue font) and moR-421-5p (magenta fonts). Sequences of miRs and their adjacent moRs may overlap in few terminal bases (grey font). Vertical bars connecting the miRBase annotated miR-421-3p and the newly predicted miR-421-5p bases indicate the complementary miRNA sequence predicted by miR&moRe2, according to the precursor folding.

MiR&moRe2 considers a preliminary read processing step in which adapter sequences are trimmed from the raw reads (Figure 1a). Trimmed reads are subsequently filtered according to (i) minimal and maximal length, (ii) minimal average read quality, and (iii) maximum two bases with low quality (Phred < 20) within the read.

Reads passing the preliminary filters (quality reads) are collapsed into unique sequence tags. The read count of each sequence tag is used to filter out sequences with low count (default < 10 reads) in order to remove possible sequencing background noise. Selected tags are exactly aligned to the reference genome. At this stage, novel miRNA precursors can optionally be predicted using the core algorithm of miRDeep2 [25], which was integrated into miR&moRe2. A common problem of sRNA-seq data alignment is that short reads can align to multiple loci in the genome, impeding the identification of their actual transcription origin. Moreover, miRNAs expressed from multiple loci [26], which share common sequences, would be disregarded if solely uniquely aligned reads were considered. To control multiple alignments, miR&moRe2 discards the reads mapping to too many loci (default > 5) outside known and predicted miRNA precursor genes.

MiRNA precursor sequences are extracted from the reference genome according to their annotation coordinates plus 30 nucleotides flanking the precursor ends to compose “extended” miRNA precursors. The quality reads are then aligned to the extended precursors allowing up to two mismatches. Alignments with no mismatches are then processed to identify non-overlapping alignment blocks, which are scanned to (i) define which mature miRNAs are expressed, (ii) to predict moRNA coordinates, and (iii) to discover new miRNAs that were not annotated in precursor genes. Novel miRNAs are inferred from the known miRNA complementary sequence projected on the precursor, according to the secondary structure hairpin-like folding. MoRNA coordinates in the precursor are inferred from the read blocks aligned to the precursor flanking sequences (Figure 1b). Moreover, employing a conservative approach, sequences that are shared with miRs are filtered out from moRNAs. 

Once up to four sRNAs expressed from each known or predicted precursor have been defined, all the alignments are parsed to identify and quantify miRNA and moRNA variants (isomiRs and isomoRs). Alignments with two mismatches are further filtered to keep only those with the two mismatches at the 3′, in order to account for post processing nucleotide addition to mature miRNAs [27,28]. In the current implementation, miR&moRe2 discriminates sRNA variations according to the number of mismatched bases (one or two mismatches) and length (shorter or longer), which are further distinguished according to the variation position in the sRNA (at the 5′, 3′, or both for length variations), overall classifying seven different isoform types. 

IsomiRs and isomoRs expression estimates are collected in a table and used to compute the overall expression for each sRNA as read counts.

MiR&moRe2 allows the user to adjust several parameters to control the various filtering steps and the read assignment rules, ultimately modulating the stringency of the predictions and expression estimate.

### 2.2. MiR&moRe2 Recovers Known MoRNAs

To evaluate miR&moRe2 performance, we applied our method to three datasets from previous studies on human cell samples in which (i) moRNAs were detected, validated and studied (ASI dataset) [14], (ii) moRNAs were considered and explicitly mentioned (BUR dataset) [20], and (iii) sequences derived from miRNA precursor flanking regions were detected (MAV dataset) [29] (Table 1).

Considering the datasets together, ~8% of miRNA-expressing precursors expressed at least one moRNA. The new sRNAs identified by miR&moRe2 contributed only < 1% on average to sample expression, proving that miR&moRe2 can detect sRNAs represented by few reads. Besides, moRNA expression represented up to 6% (Figure S1) in the MAV samples that sequenced only the cell nuclear fraction, confirming that moRNAs are particularly enriched in the nucleus [11].

**Figure 2:**
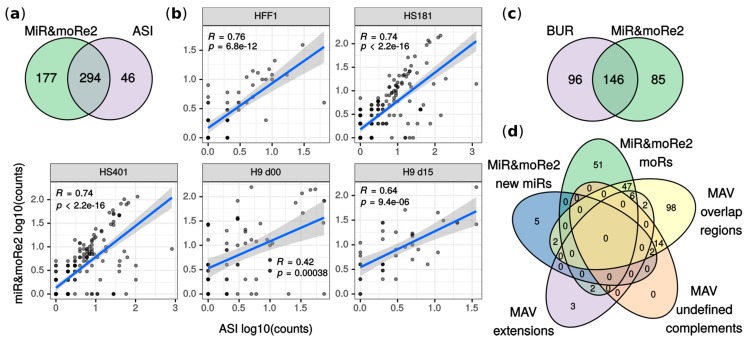
Comparison between original work results and miR&moRe predictions. (**a**) MoRNAs detected by miR&moRe2 from the ASI dataset overlap to moRNAs reported in [14]; (**b**) correlation of moRNA expression estimates (read counts) between miR&moRe2 predictions and values in [14] for each sample of the dataset (human embryonic stem cell lines HS181, HS401 and H9, plus fibroblast HFF-1). H9 d00/d15: day 0 and day 15 of H9 differentiation as detailed in [14]; (**c**) moRNAs reported in [20] compared to miR&moRe2 moRNAs predicted from the BUR dataset; (**d**) miR&moRe2 moRs and new miRs predicted from the MAV dataset, compared to miRNA gene aligned sequences grouped as in [29] (overlap-regions, undefined complement and extension sequences).

MiR&moRe2 detected ~86% of moRNAs previously reported in ASI (Figure 2a). Among them, moR-103a-2-3p was validated and further shown to enforce the flanking miRNA regulative function by Asikainen et colleagues [14]. Moreover, the moRNA expression estimated by miR&moRe2 positively correlated with the originally reported values (Figure 2b). In addition, 13 previously reported moRNAs were amongst sequences unassigned by miR&moRe2 due to its stringent criteria for the sequence assignment to the miRNA precursor sRNAs. Indeed, by using less stringent parameter settings (see Methods), miR&moRe2 identified one additional moRNA previously reported by Asikainen et colleagues. Further, correlation of expression estimates increased by ~0.02 in HS181, HS401 and HFF-1 samples, and by 0.19 in H9 d00 sample. Conversely, H9 d15 sample showed 0.03 reduced correlation which, nevertheless, was significantly positive (Figure S2). This analysis shows that miR&moRe2 approach is conservative, yet flexible enough to modulate its predictions.

MiR&moRe2 predictions in BUR and MAV datasets showed respectively ~60% and ~66% overlap with previous findings (Figure 2c,d). Mahlab-Aviv and colleagues discriminated between sequences aligned strictly to pre-miRNA flanking nucleotides (extensions), and sequences overlapping, but not perfectly matching, mature miRNAs (overlap-regions). We compared moRNAs predicted by miR&moRe2 with either extension and overlap-regions since miR&moRe2 tolerates a little moRNA overlap to the adjacent miRNA. Most of the extensions (8 out of 13) were classified as moRNAs by miR&moRe2 (Figure 2d). Conversely, most of the overlap-regions (112 out of 114) did not comply with miR&moRe2 assignment criteria and were consistently reported as unassigned sequences (Figure S3).

### 2.3. MoRNA Expression Is Impaired upon Knock-Down of the miRNA Biogenesis Pathway

Friedländer and colleagues [30] validated their prediction method by analyzing the expression pattern of the predicted pre-miRNAs and miRNAs upon silencing of the miRNA biogenesis pathway in the SH-SY5Y neuroblastoma cell line (FRI dataset; Table 1). Similarly, we applied miR&moRe2 to the FRI dataset to explore whether expression of novel miRNAs and moRNAs was affected.

Overall, 111 moRNAs and 52 new miRNAs were profiled by miR&More2 in FRI samples. Upon knocking down of the biogenesis pathway, moRNA expression was reduced in median by 40% to 70%, whereas novel miRNA expression was reduced from 44% to 70% (Table S1). 

**Figure 3:**
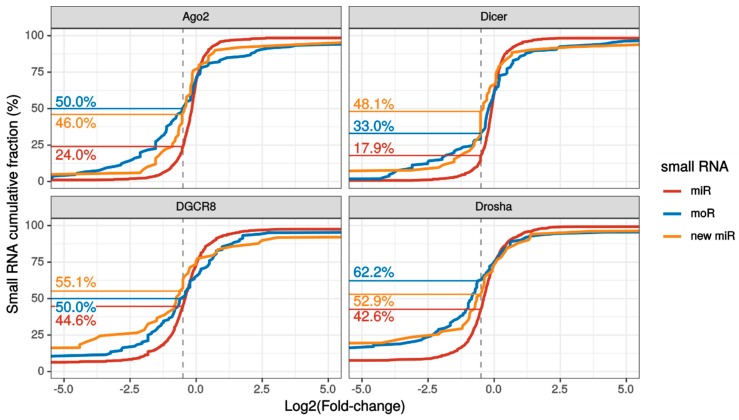
Small RNA expression variation upon knockdown of the miRNA biogenesis pathway. Cumulative fraction of known miRNAs (red lines), moRNAs (blue lines) and novel miRNAs (yellow lines) with the indicated or lower log2 fold change (LFC) with respect to control sample, upon knockdown of either Argonaute2 (Ago2), Dicer, Drosha or DGCR8 in SH-SY5Y cells. The vertical dashed grey lines indicate the LFC threshold for which the down-regulation was substantial (i.e., >30%). The fractions of substantially down-regulated sRNAs are reported on the left side, following the horizontal lines. This plot focuses on LFC between -5 and +5 to better appreciate the different slope of the cumulative lines close to the substantial downregulation LFC threshold. Plot of LFC full range values is reported in Figure S4.

Interestingly, the fraction of substantially down regulated (> 30% expression reduction) sRNAs was larger for novel RNAs than known miRNAs in each knockdown experiment (Figure 3 and Figure S4). Summing over the experiments, 96% moRNAs and 85% new miRNAs were considerably down-regulated upon silencing of the biogenesis pathway.

These results suggest that novel RNAs predicted by miR&moRe2 originated from the miRNA biogenesis process. 

### 2.4. MoRNAs Expression in Seven Human Blood Cell Populations

To illustrate miR&moRe2 discovery power and application, we estimated miRNA and moRNA expression in human cells applying miR&moRe2 to a large dataset of 297 cell population samples from human peripheral blood [31]: B-cells, cytotoxic T-cells, helper T-cells, natural killers (NK), monocytes, neutrophils, and red blood cells (RBC) (JUZ dataset; Table 1). 

**Figure 4:**
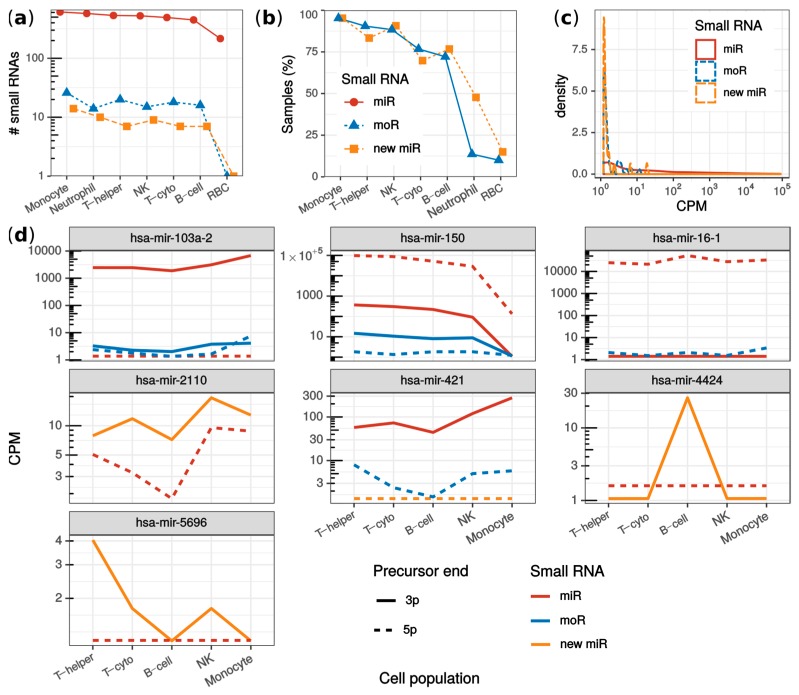
Analysis of monocytes, neutrophils, red blood cells, B-cells, helper T-cells, cytotoxic T-cells and natural killers from the JUZ dataset. (**a**) fraction of samples per cell population in which new miRNAs and moRNAs were detected; (**b**) number of sRNAs detected in each cell population grouping by miRNAs (red), new miRNAs (yellow) and moRNAs (blue); (**c**) sRNA expression (in counts per millions; CPM) distribution comparing known miRNAs (red solid line), new moRNAs (dashed yellow line) and moRNAs (blue dotted line); (**d**) moRNAs and new miRNAs with significantly varied expression among blood cell populations. Median normalized expression (CPM) is reported for moRNAs (blue lines) as well as for the other sRNAs expressed by the parent precursor transcript: miRNAs (red lines) and new complementary miRNAs (yellow lines), from either the 3′ (solid lines) and the 5′ (dotted lines) precursor ends. T-helper: helper T-cells; T-cyto: cytotoxic T-cells; NK: natural killers; RBC: red blood cells; CPM: counts per million mapped reads.

Overall, 25 new miRNAs and 38 moRNAs were detected (Figure S5). The number of detected sRNAs was similar across the cell populations, except for RBC (Figure 4a). Either new miRNAs or moRNAs were detected in 73% of the samples. Specifically, the highest detection rate was observed in monocytes, where 95% of samples presented new sRNAs (Figure 4b). In contrast, only moR-4521-5p and miR-451a-3p new sRNAs were identified in RBC, and in only 10% and 15% of RBC samples, respectively. This was explained by the highly specialized transcriptome rather than by a lower sample sequencing depth of the RBC (Figure S6). Even though with less consistency within cell population samples, moR-4521-5p was detected also in NKs, monocytes, B-cells, and neutrophils, while miR-451a-3p was detected also in monocytes (Figure S6).

A positive correlation was observed between the number of detected sRNAs and sample sequencing depth (Figure S7). To validate that this correlation was not specific to the dataset, we composed an independent dataset (LAP dataset [32], see Methods) having lymphoblastoid cell line biological replicates sequenced at different depths (from ~1 to ~30 million reads). The trend observed in the JUZ dataset was confirmed also in the LAP dataset: both moRNA and miRNA detection rate correlated positively and significantly with the sample sequencing depth (Spearman’s ⍴ = 0.8; *p*-value < 0.0031; Figure S8). Moreover, it was interesting to observe that the largest sample of the LAP dataset (~30 million reads) yielded 22 more than the total moRNAs detected in the JUZ dataset. This could be explained by the relatively low sequencing depth of the JUZ dataset samples (~0.4 to ~13 million reads). However, in blood cells, few moRNAs, such as moR-150-3p, moR-421-5p, moR-103a-2-3/5p, and moR-4424-3p were detected with consistent and sizable expression, especially in lymphoid cells (Figure S5), which suggested a cell population specificity of these moRNAs.

The new sRNAs identified in blood cells (JUZ dataset) showed generally lower expression levels than known miRNAs (Figure 4c). This observation may explain why they were disregarded by Juzenas et colleagues. Nevertheless, among the most expressed moRNAs (Table 2), we detected moRNAs already described in other studies. For instance, moR-150-3p, moR-24-2-5p, moR-421-5p, moR-21-5p, and moR-6724-5p were recently identified by us in multiple myeloma samples [17], whereas moR-103a-2-3p was validated and studied by Asikainen et al. [14].

To further illustrate possible downstream analysis allowed by miR&moRe2 results, we compared sRNA expression among cell populations using miR&moRe2 estimates. Rather than being an exhaustive analysis as performed by Juzenas and colleagues, we were interested in inspecting whether the moRNAs and novel miRNAs predicted by miR&moRe2 were differentially expressed across the different cell types. Overall, 395 sRNAs were significantly varied, including four moRNAs (moR-103a-2-5p, moR-150-3p, moR-16-1-5p and moR-421-5p) and three new miRNAs (miR-2110-3p, miR-4424-3p and miR-5696-3p; Figure 4e). Interestingly, all the differentially expressed new miRNAs were more abundant than the annotated miRNAs from the same precursor. Further, each of mir-103a-2, mir-150, mir-16-1, mir-2110, and mir-421 precursors generated multiple sRNAs with significant differential expression. For instance, mir-150 had significantly lower expression in monocytes compared to lymphoid cells for all its sRNAs except moR-150-5p, which did not result statistically significant, possibly because of its very low expression. Moreover, the similar expression pattern of miR-421-3p and moR-421-5p, together with the very low expression of miR-421-5p, intriguingly suggests that the moR-421-5p may take the place of miR-421-5p as the main sRNA originating from mir-421.

Intriguingly, our analysis of LAP data from a lymphoblastoid cell line immortalized with Epstein–Barr virus (EBV) infection detected new miRNAs and moRNAs from EBV pre-miRNAs (Figure S9). This is in accordance with previous findings on moRNAs expressed by viral precursors in infected cells [33,34].

## 3. Discussion

The advent of high-throughput RNA sequencing coupled with advanced bioinformatics analysis provided molecular biology researchers with a technology of unprecedented discovery power [35]. The recent identification of novel RNA molecules by means of innovative bioinformatics methods [36,37,38] showed that thorough RNA-seq data inspection can be rewarding. MoRNAs, in particular, were identified and further examined by a few studies performing custom analysis combined with manual curation of specific datasets [2,18,39]. Nevertheless, even though the first description of moRNAs dates back more than ten years [3], the lack of bioinformatics tools explicitly considering moRNAs may have contributed to overlook these RNAs in many studies.

In this work, we presented miR&moRe2, a novel bioinformatics tool for detection and quantification of miRNAs, moRNAs, and their isoforms, from sRNA-seq data. The former implementation of miR&moRe [6] was proven successful in applicative studies [8,13,15,17,19] but was developed as an in-house method and considered only human data. Since then, the miR&moRe pipeline has been considerably improved by adding new features. Now, miR&moRe2 can be used for any species for which a reference genome has been assembled. Moreover, it includes prediction of miRNA precursors, allowing, in turn, the identification of moRNAs derived from still unannotated precursors. Furthermore, the code was deeply revised to support updated versions of the tools included in the pipeline, as well as to increase the ease of use and the computational efficiency through parallel computing.

Regarding miR&moRe2 pipeline design and implementation features, a series of filters on raw data are applied by means of efficient methods [40], and optimal parameters for read alignment are employed [41]. Moreover, miR&moRe2 makes use of the best performing and widely used methods for miRNA prediction, miRDeep2 [25] and RNAfold [42]. Altogether, these implementation strategies aimed at reducing false predictions derived from poor quality sequencing data.

We applied miR&moRe2 to public datasets from three previous studies (ASI, BUR, and MAV) reporting on moRNAs. In the first study (ASI) [14] moRNAs were specifically reported along with their expression estimates. In contrast, the second work (BUR) [20] detected moRNAs and reported them using conventional naming [43]; whereas in MAV [29] moRNAs were referred with a custom denomination. Straightforward comparisons were not possible since each work applied its own custom discovery and analysis pipeline, which, in addition, were based on currently outdated miRNA annotation. Moreover, the authors did not provide automated software to replicate their analysis, and, in one case [29], they used custom naming to refer to moRNAs. These issues would not have arisen if the authors could have used an automated bioinformatics pipeline such miR&moRe2. We speculate that by tuning miR&moRe2 parameters, for instance regarding read pre-processing and alignment, our method could identify additional moRNAs to increase the match with previous works. However, given the lack of a gold standard dataset for moRNA validation, achieving a perfect match with other authors’ findings was not the aim of this study. Nevertheless, we observed significant overlap between the original works’ and miR&moRe2 results, which supported the reliability of our method’s findings. Moreover, novel moRNAs were detected from the analyzed data.

In accordance with previous reports [11,20,29], moRNAs were more abundant in the nuclear fraction of cellular RNA. Further, moRNAs were generally less expressed than miRNAs, but specific moRNAs were abundant, even more than the flanking miRNA [11], representing an alternative product respective to the mature miRNA from the same hairpin arm.

To further evaluate miR&moRe2 predictions, we analyzed the FRI dataset [30] in which the miRNA biogenesis pathway was silenced at different stages, and we observed that moRNAs and new miRNAs identified were downregulated similarly to the known miRNAs. Beyond indirectly validating our method’s predictions, this supports previous hypothesis that moRNA and miRNA biogenesis are linked [11].

To show that moRNA expression has been disregarded in previous studies, we applied miR&moRe2 on sRNA-seq data from a sizeable set of blood cell population samples from different healthy donors (JUZ dataset) [31], providing the first large-scale comparative moRNA expression analysis. Tens of moRNAs and new miRNAs were detected, albeit with lower abundance than known miRNAs. However, consistent expression in cell populations of few moRNAs, such as moR-150-3p, moR-421-5p, and moR-103a-2-3/5p, suggests that they could be a constitutive part of the normal blood cell transcriptome. This last analysis was intended simply to illustrate the possibilities enabled by miR&moRe2, including the re-analysis of many datasets available in RNA-seq repositories. Nonetheless, our results set the basis for further investigation on the novel sRNAs predicted by miR&moRe2 in blood cells.

Unlike other sRNA-seq analysis tools [2], miR&moRe2 allows a comprehensive characterization of the sRNAs generated by known and predicted miRNA precursors by detecting and quantifying the expression of both miRNAs and moRNAs with homogeneous criteria.

As performed in our earlier studies [15,17,19], the sRNA characterization performed by miR&moRe2 allowed a comprehensive evaluation of miRNA and moRNA differential expression. Interestingly, moRNAs with significantly varied expression levels among cell populations were identified. MoR-150-3p, resulting highly expressed in lymphocytes, was previously validated, confirming its high expression in B-cells and plasma cells [17]. MoR-103a-2-3p, with high expression in the present study, was previously found very abundant in JAK2 mutated cancer cells [13], overexpressed in stem cells, and functionally linked to its flanking miRNA [14]. These results underline that accounting moRNAs in sRNA comparative analysis can enrich the findings. Similar to miRNAs, moRNAs could have pleiotropic effects or act as fine tuners and they were hypothesised to cooperate with miRNAs to enhance miRNA function [14]. For these reasons, researchers should not disregard moRNA expression.

MoRNAs were shown to be expressed in different species, from ascidian to mammals, and also from viral genomes [33,34]. Our sample analyses illustrated that miR&moRe2 can be applied to human data also to obtain a metatranscriptomic profiling.

In conclusion, we demonstrated that miR&moRe2 is a valid bioinformatics tool to comprehensively analyze all the currently known sRNAs that can originate from each miRNA precursor gene. Using miR&moRe2 for sRNA analysis projects can contribute to increasing our knowledge of moRNAs and to the understanding of non-coding sRNA biogenesis and function.

## 4. Materials and Methods

### 4.1. MiR&moRe2 Implementation Details

MiR&moRe2 runs on Linux platform and is implemented with Python3, R and Bash scripts with a modular approach. The modules are linked with Scons (https://scons.org), a tool originally implemented to manage the building of complex software. Scons is a flexible tool that can be leveraged beyond software building [36] since the definition of the building steps only requires Python template scripts defining the tasks’ mutual dependencies. Then, the Scons engine scans the scripts and schedule the execution of each task, so that tasks not depending on each other (i.e., tasks that do not need sequential execution) can be run in parallel. This particular feature allows an automated and computationally efficient execution of the various steps of the analysis pipeline, as well as the possibility of analyzing multiple samples in parallel.

The most relevant software integrated in miR&moRe2 follow: adapter sequence trimming is carried out by means of Cutadapt v2.5 [40]; Bowtie v1.1.2 [44] is used to align reads to the reference genome and to miRNA precursors. The -v 0 and -k 20 parameters of Bowtie are set for whole genome alignments, whereas for alignment to extended precursors, the parameters -n 2 -l 26 -e 70 --best --strata -a -y allow optimal mapping. MiRDeep2 v0.1.2 [25] was included in miR&moRe2 to predict novel miRNA precursors. Instead, to define novel miRNAs from known pre-miRNAs, the extended precursor folding, computed with the RNAfold utility from the ViennaRNA v2.4.14 package [42], is processed with custom scripts. Other software libraries and tools used in miR&moRe2 are bedtools v2.27 [45], samtools v1.3.1 [46], FastQC v0.11.8 (http://www.bioinformatics.babraham.ac.uk/projects/fastqc/), HTseq v0.11.2 [47], and the data.table v1.12.2 R package.

MiR&moRe2 source code can be downloaded from https://github.com/egaffo/mirandmore2.

### 4.2. Dataset Features and Accession Numbers

We selected from the Sequence Read Archive (SRA) [48] sRNA Illumina sequencing datasets of human tissues or cell lines having data in FASTQ format. The accession numbers with the study reference, acronym used in this paper, and cell are listed in Table 1.

The LAP dataset was composed of 13 samples selected among sRNA-sequencing of 465 lymphoblastoid cell lines from the 1000 Genomes [32]. To obtain a homogeneous set characterized by samples of different sequencing depths, we choose samples from the HMGU sequencing center and of GBR population of origin.

### 4.3. MiR&moRe2 Parameters and Expression Analysis

Human genome sequence (H. sapiens, NCBI GRCh38) and the GRCh38_no_alt precompiled genome index were downloaded from the Bowtie website, which also included the Epstein–Barr virus genome.

The miRNA annotation in GFF3 format was downloaded from miRBase v22, from which human and EBV miRNAs were extracted and merged into one single annotation file.

Previously published results compared to miR&moRe2 predictions are in Supplementary Table S3, Table S4 and Table S7 from [14,20,29], respectively. Comparisons were performed considering only miRNAs commonly annotated between different miRBase versions. Pre-miRNA identifiers were used when mature sRNA names were not available to compare the previous work data.

To achieve maximum sensitivity of detection the following miR&moRe2 parameters were set differently from default values: MIN_COUNT=1, MIN_MORNA_LEN=15, MAX_LEN_FILTER=31, MEAN_QUAL_FILTER=26. For the additional analysis of ASI dataset, moRNA sequence filter was disallowed by setting MORFILTER from ‘conservative’ (default) to ‘permissive’, and a larger read alignment overhang with respect to the predicted moRNA coordinates was allowed by increasing the ALLOWED_OVERHANG parameter value from 3 (default) to 4.

Adapter sequence for JUZ and LAP datasets was TGGAATTCTCGGGTGCCAAGG; ATCTCGTATGCCGTCTTCTGCTTG for BUR; and TCGTATGCCGTCTTCTGCTTGT for ASI and FRI. The MAV dataset reads already were trimmed from the adapter, so we set ‘True’ the NOADAPTER miR&moRe2 parameter.

The FRI dataset was analyzed as in [30]. Small RNAs were considered only if having at least 30 reads, computed as the sum of reads mapping from the control and the knock-down datasets. Read counts were normalized by the sample sequencing depth. Fold-changes (FC = number of reads mapping from knocked down sample/number of reads mapping from control sample) were estimated comparing the following datasets: Dicer knockdown versus control 2; DGCR8 knock-down replicate 1 and 2 (pooled) versus control 1; Drosha knockdown replicate 1 and 2 (pooled) versus control 1; and Ago2 knockdown versus control 2. For estimating the number of sRNAs that were overall down-regulated by 30% or more upon knock-down of the miRNA biogenesis pathways, the two controls were pooled and compared with the four pooled knockdowns.

For the JUZ dataset analysis, loop sequences were removed from the miR&moRe2 expression matrix prior to expression normalization, which was performed according to the TMM-with-singleton-pairing method from edgeR v3.26.5 [49].

Given the small number of new miRNAs and moRNAs detected, RBC and neutrophil samples were not considered in differential expression analysis. Differential expression was assessed by means of DESeq2 v1.24.0 [50] using a likelihood ratio test considering the confounding effect given by individuals in the model, and independent filtering. *p*-values were corrected with Benjamini-Hochberg method and significance threshold was ≤ 0.1.

### 4.4. Additional Software and Packages

All the analyses were performed on Ubuntu Server 16.04.06 LTS Linux platform. The extended hsa-mir-421 secondary structure in Figure 1b was computed with the RNAfold utility of the ViennaRNA v2.4.14 package [42]. Other software used in this work were Python v3.5.2, R v3.6.2 and Rstudio Server v1.2.5033, and the R packages pheatmap v1.0.12, limma v3.40.2 [51], ggplot2 v3.2.0 [52], viridis v0.5.1, and VennDiagram v1.6.20 [53].

## Figures and Tables

**Table 1:** Small RNA-seq datasets considered in this work.

Acronym	Cell of Origin	Reference	SRA IDs
ASI	hESC, fibroblasts	Asikainen et al. 2015	SRR1616134-36
			SRR026761-62
BUR	HeLa	Burroughs et al. 2012	DRR001488-89
MAV	HeLa	Mahlab-Aviv et al. 2018	SRR6155355-58
			SRR5804909-14
FRI	SH-SY5Y	Friedländer et al. 2014	SRR952248-49
			SRR952288-89
			SRR952290
			SRR952309-11
JUZ	Monocytes, neutrophils, red blood cells, helper T-cells, cytotoxic T-cells, B-cells, natural killers	Juzenas et al. 2017	SRR5755813-6109
LAP	Lymphoblastoid cell line cells	Lappalainen et al. 2013	ERR187515
			ERR187573
			ERR187587
			ERR187595
			ERR187647
			ERR187758
			ERR187761
			ERR187786
			ERR187791
			ERR187813
			ERR187918
			ERR187922
			ERR204769

**Table 2:** The 15 most expressed moRNAs and new miRNAs in JUZ dataset. Normalized expression given as average counts per million mapped reads.

Small RNA	B-Cell	Natural Killer	Cytotoxic T-Cell	Helper T-Cell	Monocyte	Neutrophil
miR-2110-3p	11.0	29.5	17.8	13.3	17.6	22.8
moR-150-3p	16.2	12.6	17.6	20.2	1.2	1.2
moR-421-5p	4.1	10.7	6.8	12.8	7.9	1.4
miR-4424-3p	33.5	1.2	1.2	1.2	1.2	1.2
moR-103a-2-3p	4.5	7.5	5.7	5.5	7.0	1.2
moR-103a-2-5p	1.4	2.7	4.6	4.4	7.7	1.5
moR-150-5p	10.5	3.6	1.9	3.0	1.2	1.2
moR-16-1-5p	6.6	1.9	1.5	2.5	4.6	1.3
moR-24-2-5p	3.9	5.3	1.6	2.8	3.5	1.2
moR-7-1-5p	1.7	2.7	4.1	4.8	2.0	1.2
miR-5696-3p	1.3	2.7	2.6	5.4	1.2	1.2
moR-21-5p	1.2	1.6	1.4	2.0	1.3	2.8
miR-3648-1/2-3p	2.8	1.4	1.6	1.9	1.2	1.2
moR-876-5p	1.2	3.5	1.2	1.2	1.2	1.2
moR-27a-5p	1.2	1.2	1.2	1.3	3.0	1.2

## Supplementary Materials

Supplementary materials can be found at https://www.mdpi.com/1422-0067/21/5/1754/s1. References [29,30,31,32] are also cited in Supplementary Materials.

## Abbreviations

miRNA	microRNA
moRNA	miRNA-offset RNA
sRNA	small RNA
RNA-seq	RNA sequencing
sRNA-seq	small RNA sequencing
SRA	Sequence Read Archive
CPM	Count Per Million mapped reads
LFC	Log_2_ Fold Change
