# Supplementary material for: MiR&moRe2: A Bioinformatics Tool to Characterize microRNAs and microRNA-Offset RNAs from Small RNA-Seq Data
Source: Int J Mol Sci. 2020 Mar 4;21(5):1754. doi: 10.3390/ijms21051754 (PMC7084216; doi:10.3390/ijms21051754)
Supplement: Supplementary file 1 [file ijms-21-01754-s001.pdf]

Supplementary Material

MiR&moRe2: a bioinformatics tool to characterize microRNAs and microRNA-offset RNAs from small RNA-seq data

Table of contents

Supplementary Figures **2**

Supplementary Tables **6**

References **7**

Supplementary Figures

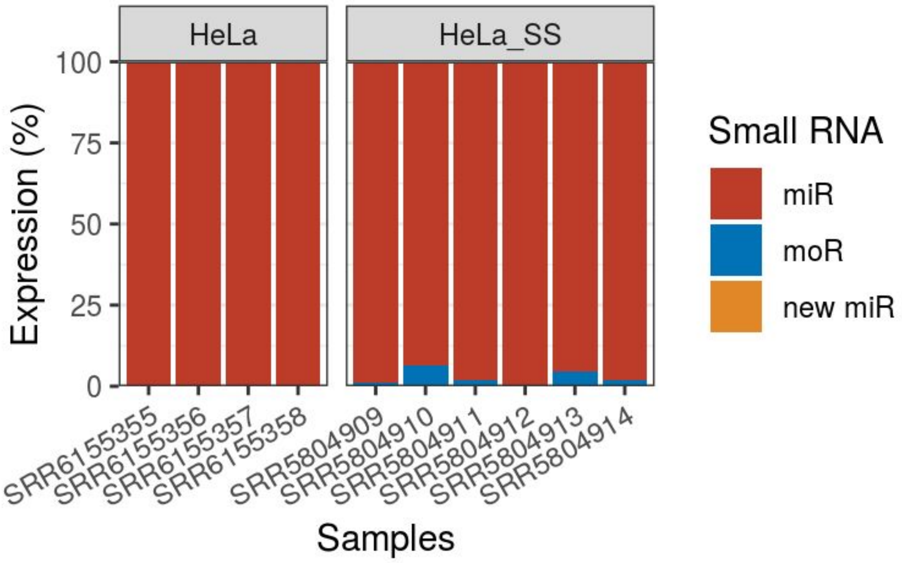

Supplementary Figure S1. MAV dataset (Mahlab-Aviv et al. 2018) sample expression contribution of small RNAs identified by miR&moRe2 in HeLa cells and the HeLa supraspliceosome nuclear fraction (HeLa_SS).

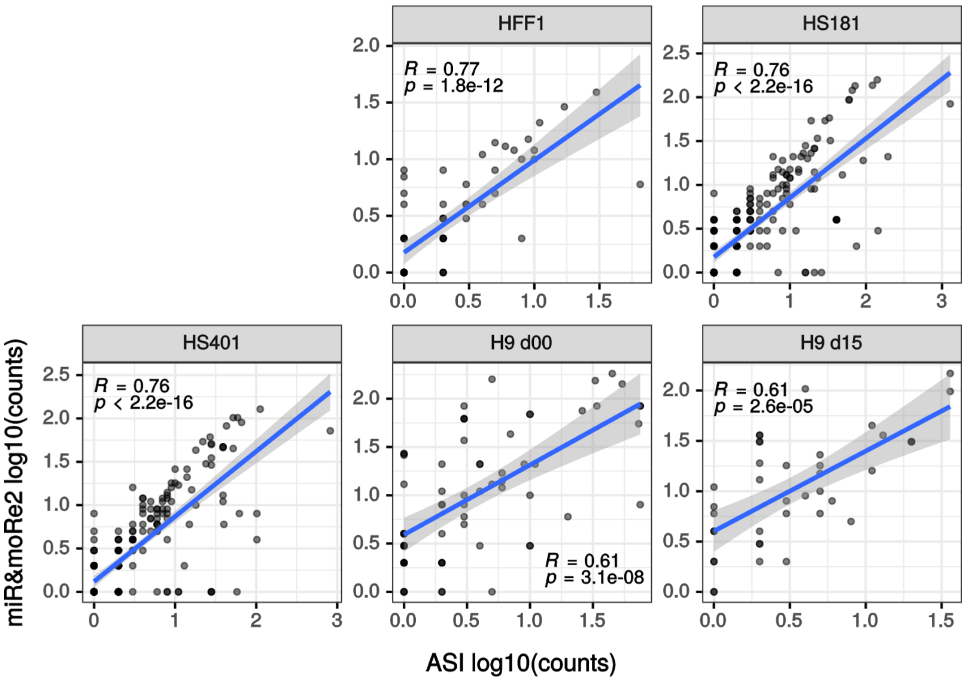

Supplementary Figure S2. MiR&moRe2 moRNA expression estimates (read counts) using parameter values less stringent than defaults (ALLOWED_OVERHANG = 4; MORFILTER = permissive) in samples of the ASI dataset (hESC cell lines HS181, HS401 and H9, plus fibroblast HFF-1), compared to values originally reported by Asikainen et al. [14]. H9 d00/ d15: day 0 and day 15 of H9 differentiation as detailed in [14].

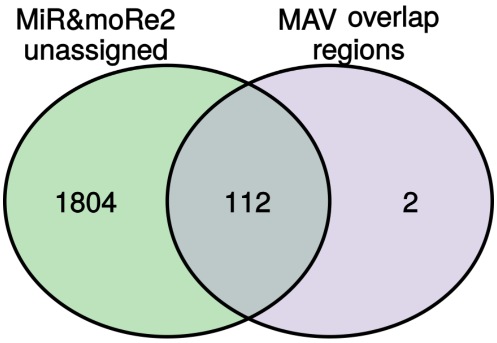

Supplementary Figure S3. Venn diagram of overlaps between miR&moRe2 unassigned sequences and the MAV dataset (Mahlab-Aviv et al. 2018) "overlap regions" that were not categorized by miR&moRe as either moRNAs or new miRNAs.

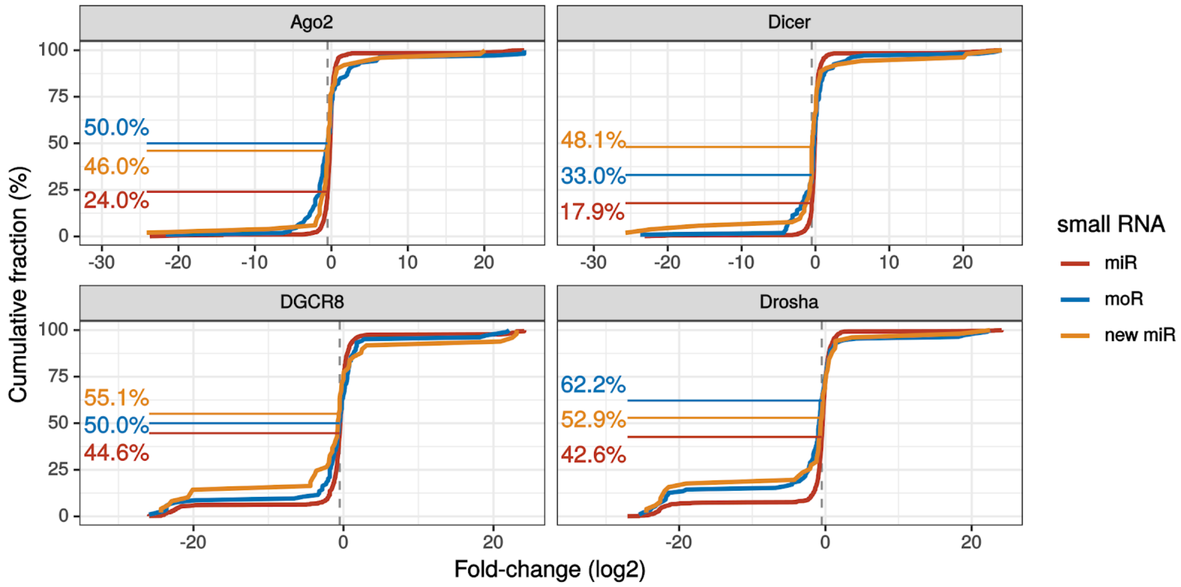

Supplementary Figure S4. Cumulative fraction of known miRNAs (red lines), moRNAs (blue lines) and novel miRNAs (yellow lines) with the indicated or lower fold change (FC) with respect to control sample upon knockdown of either Argonaute2 (Ago2), Dicer, Drosha or DGCR8 in SH-SY5Y cells. Vertical dashed grey lines indicates the threshold for which down-regulation was substantial (>30%; -0.5 log2FC), and the fractions of substantially down-regulated sRNAs are reported on the left side.

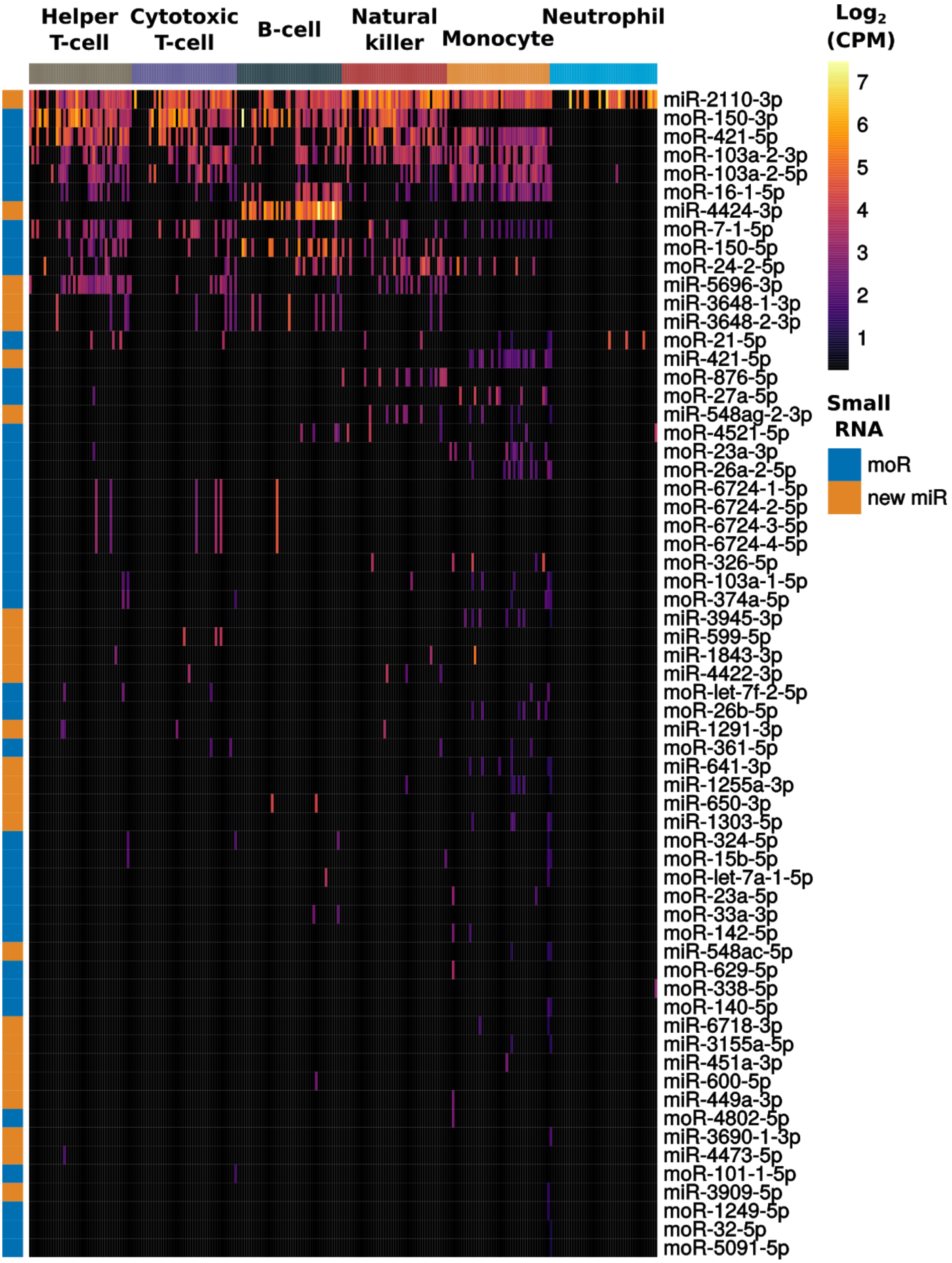

Supplementary Figure S5. Heatmap of moRNA expression ($\log_2$(CPM)) in blood cells from the Juzenas dataset. MoRNAs are ordered according to descending overall mean expression.

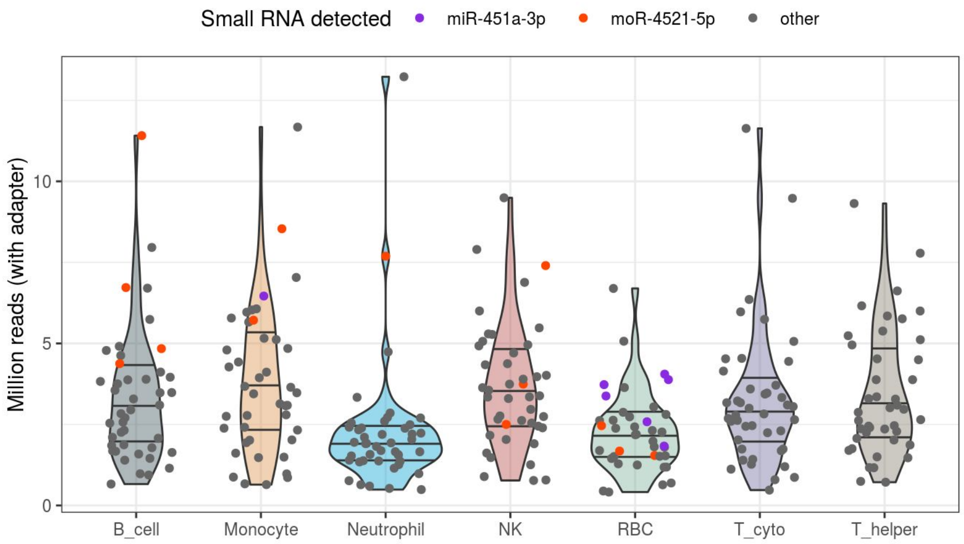

Supplementary Figure S6. Violin plot of sample sequencing depth in the JUZ dataset. Dots represent samples, displayed according to cell population (horizontal axis) and amount of adapter ligated sequenced reads (vertical axis). Samples in which moR-4521-5p and miR-451a-3p were detected are colored in orange and violet, respectively.

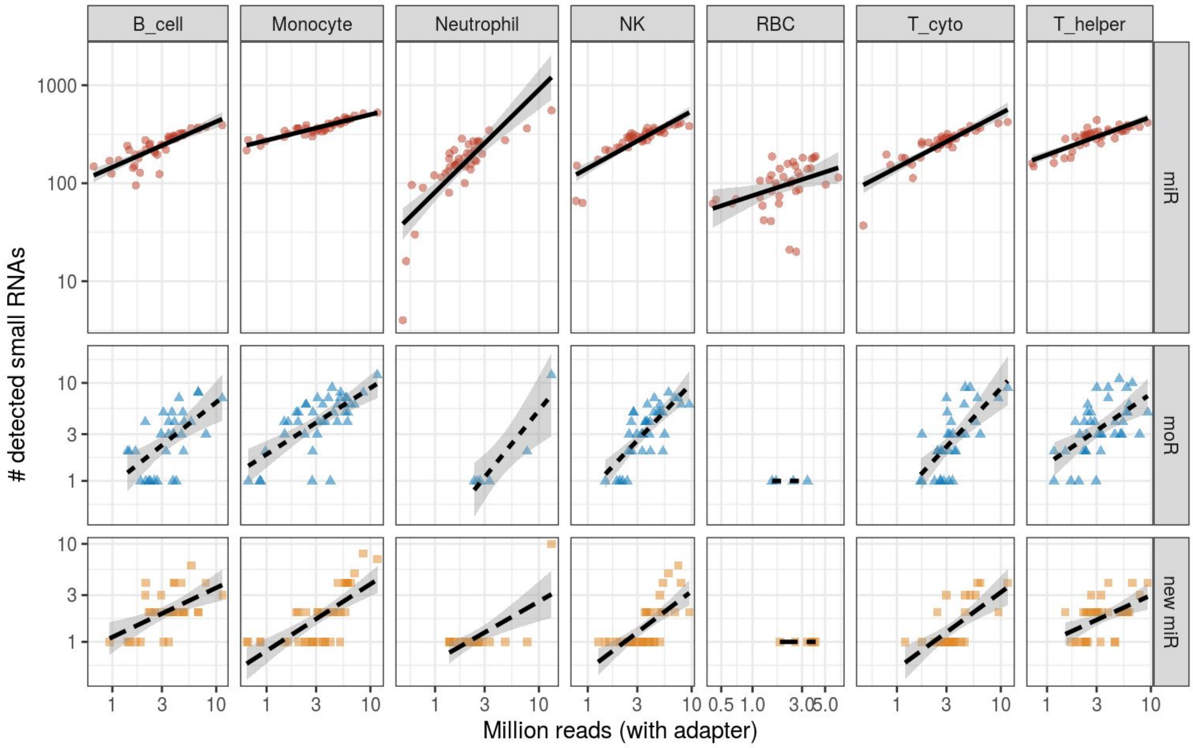

Supplementary Figure S7. MiR&moRe2 detected small RNAs from the JUZ dataset (Juzenas et al. 2017). Correlation of small RNA detection rate (vertical axis) and sample sequencing depth (horizontal axis), for each cell population (columns) and small RNA class (rows).

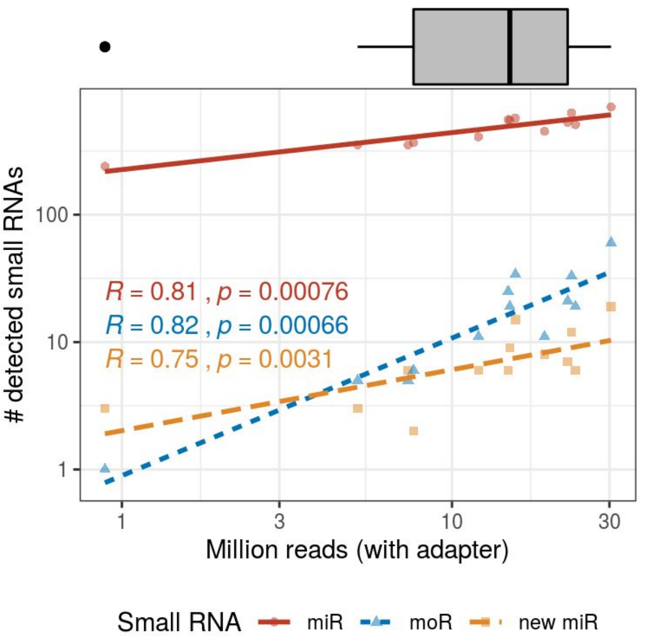

Supplementary Figure S8. Correlation of small RNA type and sample sequencing depth for each sample from 13 samples from the LAP dataset (Lappalainen et al. 2013). Samples were selected based on their sequencing center (HMGU) and population of origin (GBR). The boxplot on top represents the samples' sequencing depth distribution.

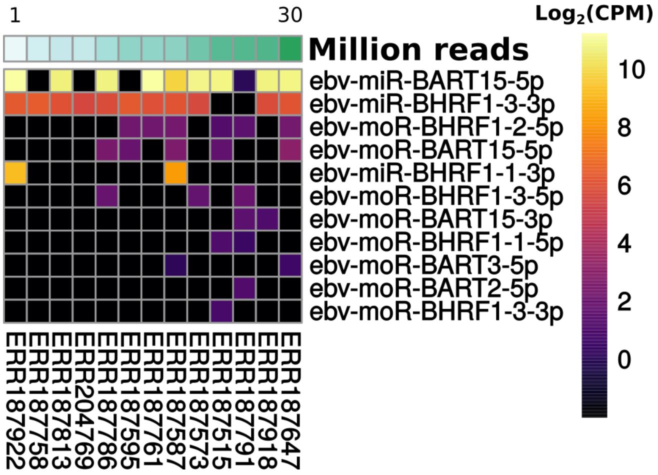

Supplementary Figure S9. New miRNAs and moRNAs from EBV pre-miRNAs detected in the LAP dataset (Lappalainen et al. 2013).

Supplementary Tables

Supplementary Table S1. Median down-regulation, with respect to control samples, of small RNAs detected by miR&moRe2 in SH-SY5Y cells (Friedländer et al. 2014), upon knockdown of either DGCR8, Drosha, Dicer or Argonaute2 (Ago2).

	**DGCR8**	**Drosha**	**Dicer**	**Ago2**
MiRNAs	63.65%	66.37%	81.75%	78.57%
MoRNAs	40.41%	41.19%	70.08%	45.78%
New miRNAs	43.52%	52.39%	69.83%	66.86%

References

Friedländer, Marc R., Esther Lizano, Anna J. S. Houben, Daniela Bezdan, Mónica Báñez-Coronel, Grzegorz Kudla, Elisabet Mateu-Huertas, et al. 2014. "Evidence for the Biogenesis of More than 1,000 Novel Human microRNAs." *Genome Biology* 15 (4): R57.

Juzenas, Simonas, Geetha Venkatesh, Matthias Hübenthal, Marc P. Hoeppner, Zhipei Gracie Du, Maren Paulsen, Philip Rosenstiel, et al. 2017. "A Comprehensive, Cell Specific microRNA Catalogue of Human Peripheral Blood." *Nucleic Acids Research* 45 (16): 9290–9301.

Lappalainen, Tuuli, Michael Sammeth, Marc R. Friedländer, Peter A. C. 't Hoen, Jean Monlong, Manuel A. Rivas, Mar Gonzàlez-Porta, et al. 2013. "Transcriptome and Genome Sequencing Uncovers Functional Variation in Humans." *Nature* 501 (7468): 506–11.

Mahlab-Aviv, Shelly, Ayub Boulos, Ayelet R. Peretz, Tsiona Eliyahu, Liran Carmel, Ruth Sperling, and Michal Linial. 2018. "Small RNA Sequences Derived from Pre-microRNAs in the Supraspliceosome." *Nucleic Acids Research* 46 (20): 11014–29.
